# Systemic analysis of the response of *Aspergillus niger *to ambient pH

**DOI:** 10.1186/gb-2009-10-5-r47

**Published:** 2009-05-01

**Authors:** Mikael R Andersen, Linda Lehmann, Jens Nielsen

**Affiliations:** 1 Center for Microbial Biotechnology, Department of Systems Biology, Technical University of Denmark, DK-2800 Kgs. Lyngby, Denmark; 2 Current address: Department of Chemical and Biological Engineering, Chalmers University of Technology, SE-412 96 Gothenburg, Sweden

## Abstract

**Background:**

The filamentous fungus *Aspergillus niger *is an exceptionally efficient producer of organic acids, which is one of the reasons for its relevance to industrial processes and commercial importance. While it is known that the mechanisms regulating this production are tied to the levels of ambient pH, the reasons and mechanisms for this are poorly understood.

**Methods:**

To cast light on the connection between extracellular pH and acid production, we integrate results from two genome-based strategies: A novel method of genome-scale modeling of the response, and transcriptome analysis across three levels of pH.

**Results:**

With genome scale modeling with an optimization for extracellular proton-production, it was possible to reproduce the preferred pH levels for citrate and oxalate. Transcriptome analysis and clustering expanded upon these results and allowed the identification of 162 clusters with distinct transcription patterns across the different pH-levels examined. New and previously described pH-dependent *cis*-acting promoter elements were identified. Combining transcriptome data with genomic coordinates identified four pH-regulated secondary metabolite gene clusters. Integration of regulatory profiles with functional genomics led to the identification of candidate genes for all steps of the *pal/pacC *pH signalling pathway.

**Conclusions:**

The combination of genome-scale modeling with comparative genomics and transcriptome analysis has provided systems-wide insights into the evolution of highly efficient acidification as well as production process applicable knowledge on the transcriptional regulation of pH response in the industrially important *A. niger*. It has also made clear that filamentous fungi have evolved to employ several offensive strategies for out-competing rival organisms.

## Abstract 2

Systems modeling of *Aspergillus niger* under different pH conditions reveals novel pH-regulated metabolic genes and signaling genes in the pal/pacC pathway.

## Background

The subject for much discussion has been why *Aspergillus niger *produces organic acids in the amounts of which it is capable of. If *A. niger *is grown in an unbuffered medium, it will fairly quickly acidify the medium to a pH below 2. Production processes with cultivation of *A. niger *can convert as much as 95% of the available carbon to organic acids, making it a viable process for producing bulk chemicals [1]. The evolutionary strategy behind this trait remains obscure, but one of several hypotheses suggests that the secretion of acids helps degrade the plant cell walls on which the saprotrophic fungus thrives, that it slows the growth of competing organisms, and that the organic acids chelate sparse trace metals and make them available to the fungus [2].

The production of organic acids by *A. niger *has been shown in several studies to be dependent on ambient pH. Oxalic acid production is most efficient at pH 5 to 8 and is completely absent below pH 3.0 [3]. Gluconic acid production is optimal at pH 5.5, but it is found at all levels of pH from 2 through 8 [4,5]. Citric acid production begins at pH 3.0 and is optimal just below pH 2.0 [1,6]. This suggests that an evolutionary process has selected for production of a given acid at different pH values. In this context, the work of Ruijter *et al. *[3] is interesting. They showed that a mutant strain of *A. niger*, deficient in producing gluconic acid and oxalic acid, produces citric acid at an optimum pH of 5 and without the demand for an Mn^2+^-deficient medium, which is normally essential for the production of citric acid. This suggests that the aforementioned evolution of acid production has resulted in a sophisticated system of preferred acids as a function of ambient pH, which even ensures that another acid is produced when conditions are unfavorable for production of the preferred acid. To improve our understanding of these systemic behaviors, we have adopted a genome-scale-based strategy founded on the integration of multiple types of genome-wide data ('omics'), particularly genome-scale modeling, functional genomics, and transcriptomics.

**Figure 1:**
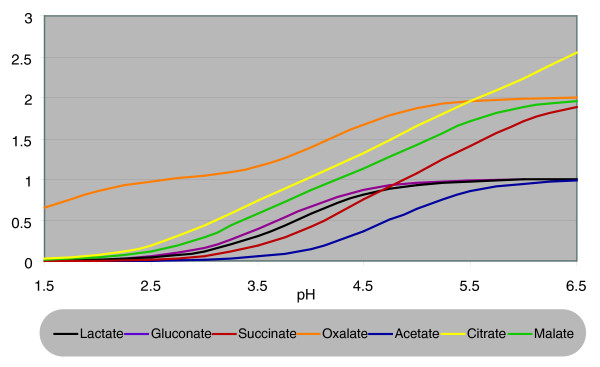
Protons per molecule of the original un-disassociated acid as a function of pH.

This approach allowed us to formulate the hypothesis that *A. niger *strives to produce - at a given pH - the organic acid that most efficiently acidifies the medium. To test this hypothesis, the model of *A. niger *metabolism presented by Andersen *et al. *[7] was expanded with reactions describing the average number of protons released from one mole of a given acid at a given pH, based on acid disassociation constants (Figure 1). This allows the use of mathematical optimization principles coupled with the knowledge of metabolic pathways, and thereby computationally determining the most efficient way of producing protons to acidify the surrounding medium as a function of pH. If these computations are in agreement with the pH dependencies of the organic acids described earlier, it will be strong evidence that *A. niger *is evolutionally optimized for acidifying its environment.

The response to ambient pH is relevant not only in the context of organic acid production. *Aspergillus niger *is an expression system for both homologous and heterologous proteins, and the expression of yield-lowering proteases has been shown to be dependent on pH [8]. Additionally, whereas processes with *A. niger *have until now been considered safe for food-grade enzyme production, a recent analysis of the *A. niger *genome [9] suggested that it may be capable of producing the carcinogenic compound fumonisin B_2_, which was confirmed by Frisvad *et al. *[10]. The carcinogen ochratoxin A has also been known to be produced by *A. niger *under certain culturing conditions [11,12]. Secondary metabolite production, such as penicillin from *Aspergillus nidulans*, has in some cases been shown to be dependent on pH [13]. Therefore, to expand on the results of the model-driven investigation of the organic acid response to pH, a physiological characterization and transcriptome analysis of triplicate cultivations at pH 2.5, 4.5, and 6.0 was made to provide a systems-wide insight into the transcriptional response to ambient pH. This allowed the identification of several genes involved in the production of organic acids reacting directly and in a coordinated manner to ambient pH.

Given that *A. niger *can grow stably at pH values ranging from below 2 to above 8 [14], it is reasonable to expect sophisticated transcriptional regulation. To use this, putative pH-dependent *cis*-acting regulatory motifs were identified. With genetic engineering of promoter regions, this may be applied to induce the production of a given gene product at the pH of the process. Another analysis was on the production of organic acids as well as identification of secondary metabolite clusters responding to pH. Furthermore, the *pacC/palABCFHI *system, a conserved fungal signal-transduction and transcriptional-regulation system, described in detail for *A. nidulans *and partially conserved in *Saccharomyces cerevisiae *[15,16], was examined, and likely orthologues were found and confirmed to have similar transcriptomic profiles in *A. niger*.

## Results

### Reproducing pH-dependent acid production *in silico*

A previously described genome-scale stoichiometric model of *A. niger *metabolism [7] was expanded, as described in Materials and methods. Acid production was simulated from pH 1.5 through 6.5 by using two different strategies: either optimization for maximal biomass production coupled to acid generation, or optimization for maximal proton generation with a fixed biomass production. The model was allowed us to use acetate, oxalate, lactate, malate, succinate, citrate, and gluconate to acidify the medium, all acids that have been observed in fermentations in our laboratory or that have been reported to be produced by *A. niger*. For each set of simulations, the acids were removed one at a time, to explore the order in which the *A. niger *simulation preferred to produce the different acids at the investigated values of pH. For complete modeling results, see Additional data files 1 and 2.

Interestingly, if all acids are included, the simulations predict oxalate as the only produced acid throughout the spectrum of pH. This is in agreement with the observation of Ruijter *et al. *[3] (and the physiological characterization in the experiments described later) that oxalate is the preferred acid in a strain capable of producing all acids.

Ruijter *et al. *[3] also reported that the production of oxalate peaks above pH 5.5, and as the calculations of Figure 1 show, this is the value at which oxalate is fully disassociated, and the value of pH at which the effect of producing oxalate for acidifying the medium levels out. The modeling results are thus in very good agreement with the hypothesis that oxalate is produced to acidify the medium, and this explains how this trait has evolved.

One could think that because oxaloacetate hydrolase - the only enzyme producing oxalic acid in *A. niger *[17] - forms 1 mole of acetate for every mole of oxalate, acetate should also appear as a product in the model simulations. However, acetate formation is not seen, meaning that the simulations predict that it is more energetically efficient to remetabolize this acetate than to use it for acidification of the medium. This is in agreement with the report by Ruijter *et al. *[3] that *A. niger *catabolizes acetate at a rate sufficient to prevent its formation during production of oxalate.

**Figure 2:**
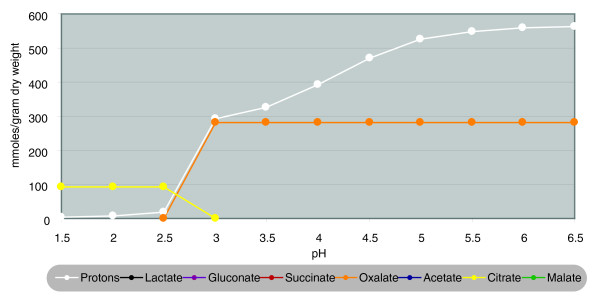
Simulated acid production with optimization criterion of maximal protons per gram of biomass. Acid disassociation was included in the model for all of the shown species, with the exception of oxalate production below pH 3.0.

However, this initial modeling did not predict how oxalate production diminishes drastically below pH 3 [3] (Pedersen *et al. *[2] reported this limit to be below pH 4), suggesting that it is due, not to inefficient acidification of the medium, but to some other factor. To simulate this, the model was adjusted to disallow medium acidification by oxalate below pH 3 (modeling results in Figure 2).

With this model, it was found that for both modeling strategies at pH levels of 1.5 to 2.5, citrate is the optimal acid for medium acidification when oxalate cannot be produced. This is the same interval used for industrial production of citric acid [1]. The necessity of the absence of oxalate production may be one reason for which very low levels of manganese are required for citrate production. Oxaloacetate hydrolase (OahA) is dependent on manganese and has a high affinity for the metal (K_*m *_for Mn^2+ ^is 4 μM [2]). Deletion of *oahA *in the work of Ruijter *et al. *[3] replicates this effect of manganese depletion, thereby inducing citrate production.

Further simulations removing proton-producing acids one by one from the model (see Additional data files 1 and 2) indicates that the pH interval of 1.5 to 2.5 is the only area where citrate is the most optimal acid, indicating how this pH preference may have evolved. Another interesting finding was that gluconate is not produced in any versions of the model unless production reactions for all other acids are removed. Because of the optimization criterion of the model, these calculations show that production of gluconate is not an energy-efficient method of acidifying the medium. It therefore seems likely that the efficient conversion of glucose to gluconate by *A. niger *has evolved not as a way of acidifying the medium, but rather as a mechanism to make rapidly glucose unavailable to competing organisms. In this context, it is interesting to note that the gluconate production is more efficient around pH 5.5, a pH level at which many fast-growing bacteria have their pH optimum.

### Physiological studies

**Figure 3:**
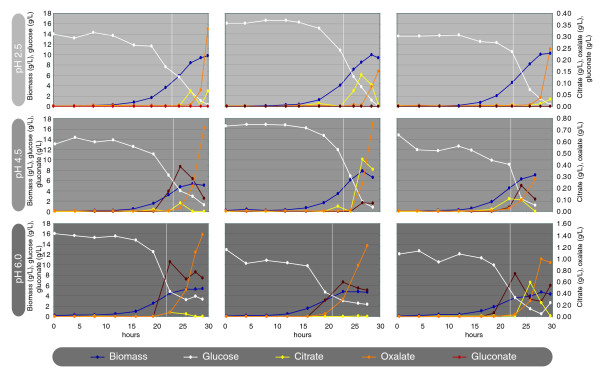
Metabolite profiles under cultivations of *Aspergillus niger *at three levels of pH. For each pH value is shown three replicates, from which biomass was sampled for transcriptome analysis. Sample times are shown with white vertical lines. Note that the pH shown in the left column is the pH at the time of sampling for transcriptome analysis. All cultures were inoculated at pH 2.5 and increased at the beginning of the growth phase if needed (see Methods for details).

To expand on the *in silico *predictions for organic acid production with *in vivo *experiments, and to gain information on other pH-dependent aspects of fungal metabolism, batch fermentations of *A. niger *BO-1 were performed in triplicates at three different pH values (2.5, 4.5, and 6.0). For each fermentation, samples were taken for determination of sugar and acid concentrations. Profiles of the cultures are shown in Figure 3. Examination of Figure 3 shows that the biomass yield decreases with increasing pH. The final biomass concentration measured decreases from 9.80 ± 0.42 g/L over 6.20 ± 1.05 g/L to 4.81 ± 0.52 g/L as pH increases (average ± standard deviation). This is due to a reciprocal increase in the produced acids. Most predominant among these is gluconate production, which is not found at all at pH 2.5, but reaches as much as 10 g/L at pH 6.0. An increase in oxalate production of roughly a factor of two also is seen with each step of pH increase. Finally, pH does not seem to have an effect on the citrate production in these cultivations. This is not surprising, as manganese was added to ensure reproducible filamentous growth for the transcriptome analysis. As Dai *et al. *[18] showed, Mn^2+ ^concentrations of 1,000 parts per billion (ppb) (the same as in the cultivation medium) ensures filamentous growth; however, this diminishes citrate production.

Additionally, citrate production is known to be limited at glucose concentrations below 2.5% [19]. Citrate concentrations are low in all batches, and the citrate-production profile is by far the least-reproducible trait across the triplicates.

From each of the nine fermentations, samples were taken for transcriptome analysis. Table 1 presents a summary of the growth and fermentation-broth composition at the time of sampling. As Table 1 shows, no significant acid production (except for gluconate production) was measured in the medium at the time of the mRNA sampling. The sampling time was chosen to be in midexponential phase, as the cell is in a reproducible pseudo-steady state at this time, thus describing pH-dependent mechanisms most reproducibly. Later sampling could result in an increased effect from extracellular acids.

**Table 1:** Sugar, acid, and biomass concentrations for *A. niger *cultivations at three levels of pH

pH	Time	μ_*max*_	Biomass	Glucose	Citrate	Oxalate	Gluconate
	(h)	(h^-1^)	(g/L)	(g/L)	(g/L)	(g/L)	(g/L)
2.5	22.77 ± 0.40	0.21 ± 0.01	4.68 ± 0.16	8.93 ± 1.84	0.01 ± 0.01	0.00 ± 0.00	0.00 ± 0.00
4.5	22.72 ± 0.63	0.21 ± 0.01	4.40 ± 0.47	7.87 ± 1.97	0.05 ± 0.05	0.02 ± 0.02	2.39 ± 3.05
6.0	21.47 ± 0.69	0.22 ± 0.02	3.71 ± 0.42	5.24 ± 1.21	0.02 ± 0.03	0.07 ± 0.05	6.36 ± 1.90

Biomass (dry weight), sugar, and acid concentrations for *A. niger *cultivations at three levels of pH at the time of sampling for transcriptome analysis. The calculated maximal specific growth rate is indicated. Values are shown as average ± standard deviations for three replicates.

### Transcriptome analysis

Samples were taken from the bioreactor cultivations for transcriptome analysis. All cultures were growing as dispersed hyphal mycelium. See Table 1 and Figure 3 for details of sampling times and conditions. Data from the three biologic triplicates at pH 2.5, 4.5, and 6.0 were statistically analyzed, and genes that are significantly regulated (Benjamini-Hochberg corrected Bayesian *P *values < 0.05) in pair-wise comparisons between two pH levels were identified.

A surprisingly large number of genes (6,228) were identified to show significant differences in transcription levels in one or more of the comparisons. As the statistical test is a very conservative one, and more than 70% of these genes are significant in more than one comparison, this high number should not be seen as a statistical artefact, but rather as a combined effect of the wide range of pH, a growth effect, and possible differences in medium composition at the time of sampling for transcriptome analysis.

**Figure 4:**
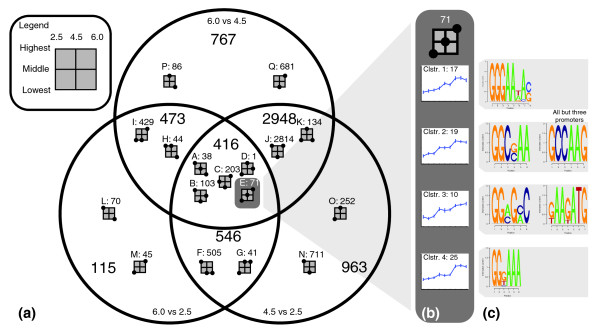
Venn diagram and clustering of genes with a significant transcriptional response to pH. The Venn diagram **(a) **is based on three pairwise comparisons. Each area in the Venn diagram is divided into subsets by the direction of the response in the different comparisons. The formation of dots in the squares shows the general tendency of the response, with the example of **(b) **having expression indices increasing with pH. Two dots on the same line means that no statistically significant difference was found between the two conditions. Each subset has been divided into clusters, as shown for the example subset (b). Predicted recognition motifs for *cis*-acting elements are shown **(c)**. Sequence logos are made as described by Schneider and Stephens [63].

To separate the effects and to identify genes for which the expression indices follow the level of pH, the regulated genes were sorted into subsets according to the direction of the statistically significant responses in the pair-wise comparisons (Figure 4a). Subsets will be referred to in the text by the letter designated in Figure 4.

Especially noteworthy is the large subset J (2,814 genes), which is upregulated at pH 2.5 and 6.0 compared with pH 4.5. It is likely that this subset is not directly regulated by pH, but rather is a part of a growth effect or a stress response, as it contains a large number of housekeeping genes, such as ribosomal subunits, DNA replication machinery, proteasome subunits, RNA-processing machinery, and so on. A GO term overrepresentation analysis (see Additional data file 3) confirmed that these terms are over-represented. The same seems to be the case for the two subsets Q and N. These subsets have the same regulatory pattern as subset J, but with one of the statistical comparisons being statistically insignificant. Therefore, clustering the genes in this manner according to the direction of the responses allows a separation of growth-related effects into subsets J, Q, and N, thus leaving the remaining subsets (2,022 genes) with a higher likelihood of being directly influenced by pH. Of these, the 109 genes in subsets A and E are of especially high interest, as these are following the levels of pH either directly (E) or inversely (A). This makes the genes in these clusters extremely likely to be regulated solely by pH and by none of the other varying factors of the cultivations.

Another point worth evaluating, when doing transcription analysis in batch cultures, is whether differing levels of glucose affect the results through glucose repression. Because the strategy of sampling at similar concentrations of biomass, to reflect the same ages of the cultures, the residual glucose concentration varies slightly between the cultures (Table 1). CreA-mediated carbon repression is well described in *A. niger *and known to be dependent on the concentration of the carbon source [20]. Genes affected by carbon repression would thus have a profile similar to those of subset I (429 genes). However, CreA is known to be autoregulated in *A. nidulans *[21], and CreA is not found to have significantly changed expression levels in any comparisons. This makes the presence of significant carbon regulation unlikely and, if present, restricted to the genes of subset I.

To examine patterns in transcription levels in the sets in more detail, a clustering algorithm was applied by using expression indices from all nine microarrays rather than averages for each group (Figure 4b). This method allowed more-detailed differentiation between the genes and the creation of clusters within each subset. In total, 162 clusters with distinct transcription patterns across the experiments were identified. An overview of the expression profiles of the clusters was made (see Additional data file 4), as well as details for each gene (see Additional data file 5).

Clustering of these genes facilitates discovery of interesting co-regulations. Especially interesting for the production of gluconic acid is the observation that the cellular membrane-bound catalase (*catR*) [22,23], is tightly co-regulated with the hydrogen peroxide- and gluconic acid-producing glucose oxidase (Gox/GodA; EC 1.1.3.4) [24,25]. Both are found in the same cluster of subset G in Figure 4. The general regulation in this subset is in good agreement with reports that oxalic acid is produced in very low amounts below pH 4.5 [4,5]. Examination of the promoter region of the genes of that particular cluster was performed to discover potential *cis*-acting elements, and two motifs were found, one being 5'-GAGGWT-3', and the other, 5'-ACRARAG-3'. The first motif is found 9 times in the promoter of *godA*, and 5 times in the *catR*-promoter, making it very likely that this motif is responsible for the co-regulation of the two genes.

Another subset of special interest to acid production and regulation by ambient pH is subset E. This subset contains three putative acid transporters, the oxalic acid-producing oxaloacetate hydrolase (*oahA*) [2,17], and the gene for a protein-regulating response from neutral to alkaline pH (PacC) [26]. Clustering of the genes places *oahA *in cluster 1 and *pacC *in cluster 3. In light of the lack of production of acetate, it is interesting that *oahA *does not seem to be co-regulated with a potential acetyl-CoA synthase or an enzyme with a similar function. This suggests that activation of acetate with CoA is not limiting for reassimilation.

As an application example of the clustering, *cis*-acting elements have been predicted for all four clusters of the subset containing *pacC *and *oahA*. Conserved motifs were found for each of the four clusters (Figure 4c), but not for the subset as a whole. A survey of subset A and the three subclusters (see Additional data file 4) showed that it was possible to find putative regulatory motifs for each of the subclusters, but not for the entire subset. That no common motif could be found for neither subset A or E supports the strategy of dividing the subsets into clusters to find truly co-regulated genes.

In an examination of the predicted motifs of subset E (Figure 4c), the second motif for cluster 2 was found to be similar to the *A. nidulans *PacC consensus-binding motif 5'-GCCARG-3' reported by Sarkar *et al. *[27]. This suggests that members of this group are regulated at least in part by PacC. PacC is known to be autoregulated in *A. nidulans *[28,29], and the motif is found in the *A. niger pacC *promoter as well. However, the clustering of *pacC *outside of cluster 2 suggests that other factors are regulating it, giving it a slightly different transcription profile from that of the members of cluster 2.

Expanding the examination of the co-regulated groups of genes, information was used on the physical location of the genes on the genomic scaffolds to find 147 clusters of genes on the genome that are colocalized as well as co-regulated. Manual inspection of the clusters allowed the identification of six putative gene clusters involved in secondary metabolite biosynthesis. Two of these were found in clusters J and Q, making them less likely to be directly regulated by pH. One of the remaining four clusters is found in subset E, cluster 2, described earlier, and contains five colocalized and co-regulated genes. A putative gibberellin-precursor synthase (Gene ID 54123) is found in this cluster.

A specific study of the three potential citrate synthases identified by Pel *et al. *[30] showed that only one is significantly regulated in any comparison: an upregulation at pH 4.5 compared with pH 2.5 (An08g10920/ID 176409). This does not correspond to a pH-dependent upregulation at pH 2.5, as would be the expected response for a citrate-synthase involved in citrate-overflow metabolism. This suggests that the pH-responsive nature of citrate production is controlled at another level (that is, transport or post-translational regulation) or that the response requires other sensing responses (manganese [18], high glucose [19], and so on) in addition to acidic pH. Based on the combined results of the modeling and the transcriptome analysis, the latter option seems to be the most likely.

Several industrially relevant proteins that are not discussed in detail here are found in the list of regulated genes, including the protease regulator PrtT, the acetate response regulator FacB/AcuB, α-amylases, and a large number of characterized and putative glucoside hydrolases, as identified by Pel *et al. *[30]. A table of the 6,228 regulated genes along with information on regulation and clustering has been compiled (see Additional data file 5).

### Data integration-based identification of the elements of the ambient pH signal-transduction pathway (*pal*) pathway in *A. niger*

It is known that proteolytic cleavage is required for activation of PacC in both *A. nidulans *[28,29,31-33] and *A. niger *[8]. Although the signal-transduction/proteolysis pathway in *A. niger *is, to our knowledge, uncharacterized, a two-step activation system for PacC is well described for *A. nidulans *(reviewed in references [15,34] and [16]).

**Figure 5:**
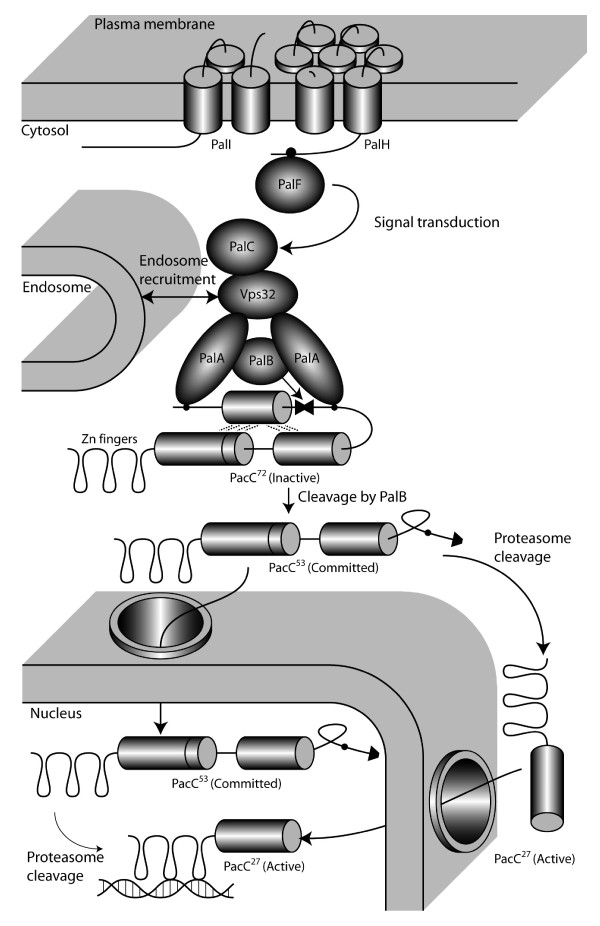
Model of pH sensing and regulation in *A. nidulans*. Black circles denote sites of protein-protein interaction, as does the overlap of two protein domains. The dotted lines of the closed conformation of PacC illustrate non-covalent interaction protecting the proteasome cleavage site. Vps32 is a part of the ESCRT-III complex that recruits to the endosome. The figure is adapted from reference [34] with information added from references [40,41,45,64].

The model of the pH-signaling transduction pathway in *A. nidulans *(Figure 5) consists of two distinct protein complexes, a plasma membrane-localized sensing complex (PalF, PalHI [35-41]) and an endosomal membrane complex (PalABC, Vps32 [42-46]), catalyzing the first proteolytic step of PacC [39,45] followed by a proteasome-catalyzed cleavage to the active form.

The *pal *pathway has been described as being 'mechanistically dissimilar to all other known eukaryotic signal transduction pathways' [44], and it is thus very likely that homologues of the *A. nidulans pal *genes in *A. niger *are indeed orthologues. A survey of the genome sequence of *A. niger *found homologues of all identified genes of the signaling pathway (Table 2).

**Table 2:** Identified pH-sensing genes in *Aspergillus nidulans *and their homologues in *A. niger*

Gene	ORFs	
	
	*A. nidulans*	*A. niger*
*palA*	AN4351	119792
*palB*	AN0256	171058
*palC*	AN7560	48740
*palF*	AN1844	Not found*
*palH*	AN6886	120044
*palI*	AN4853	52449
*vps32/snf7*	AN4240	136905
*pacC*	AN2855	47049

The *A. niger *open reading frames (ORFs) were identified by using bidirectional best blast hits.

*No hit for palF was found in the publicly available genome sequence for *A. niger *ATCC 1015, but a near-identical hit was found on the right arm of chromosome VI in the finished version of the genome sequence.

The mRNA levels of the components of the *pal *pathway are not pH regulated in *A. nidulans *[39,46], and an investigation of expression indices of the *A. niger *homologues indicates that these behave in the same manner. The putative *A. niger palA *and *palC *are not found to be significantly regulated in any comparison. *palB*, *palH*, *palI*, and *vps32 *are significantly regulated, but are found in subsets J and Q of Figure 4, which were found earlier to be more likely to be regulated by growth-dependent effects than by pH. It thus seems that the *A. niger *homologues of the *pal *pathway are independent of ambient pH as well, but may be subject to other regulation.

## Discussion

Despite the great interest in organic acid production in *A. niger* - citric and gluconic acid are bulk chemicals produced by *A. niger *processes - very little work has been published on regulation by ambient pH in *A. niger*. This study examines the response to ambient pH by the combination of results from two distinct strategies. One is a strictly hypothesis-driven application of stoichiometric modeling, with which the modeling results are compared with reported observations to test the hypothesis of *A. niger *being optimized for acidification at any given pH through the course of evolution. The other study, the transcriptomic, is a more-classic application of systems biology, in that it is a data-driven study, and the analysis both gives specific directly applicable results and allows the generation of new hypotheses on pH regulation.

The modeling part of the study showed that the optimal pH intervals for production of acids, and the types of acids produced at certain pH values, can to a certain extent be explained and simulated for citrate and oxalate, based on an assumption of an evolutionary selection for efficient acidification. The success of this approach to modeling acid production strongly suggests that *A. niger *has not evolved to outgrow its competitors such as *Escherichia coli *or to have a very efficient glucose uptake as does *Saccharomyces cerevisiae*. Instead, *A. niger *metabolism seems to be optimized to produce the most protons from the sparse nutrients available in a saprophytic environment. This also implies that acid production in *A. niger *does not stem from overflow metabolism, but rather from an objective of proton production, at least for oxalic acid and citric acid.

The inability of the model to predict the pH optimum of gluconic acid production suggests that the main objective of gluconic acid production is not related to acidification of the medium. This is supported by the detailed on-line fermentation chromatography results presented by van de Merbel *et al. *[47], in which glucose in the medium is rapidly converted fully into gluconic acid, which thereafter functions as a substrate for the rest of the fermentation. As the modeling can compute conditions only after a full degradation of the substrates, this will not show in the model. The production of gluconic acid thereby seems to be a method of making glucose unavailable to other competing organisms. This is also supported by the observations of the physiological study shown in Figure 3, in which gluconic acid produced early in the fermentation is seen to be reconsumed later.

Although the cellular response to manganese deficiency is undoubtedly complex, as the work of Dai *et al. *[18] and others have shown, it is interesting that the results of Ruijter *et al. *[3] indicate that the citrate production becomes insensitive to manganese concentrations by the deletion of glucose oxidase and oxaloacetate hydrolase. Whereas the applied model does not include the effects of manganese deficiency, it was able to replicate the effect of citrate being produced in an oxalate-deficient strain. The absence of manganese presumably has other effects that improve citrate yields, but it seems, based on these results, that one reason for its effect is the dependence of oxaloacetate hydrolase on manganese.

In modeling acid production with optimization for growth (see Additional data file 1), the biomass production increases as a function of pH. This is opposite that observed in the *in vivo *experiments. One reason for this effect is that it is very unlikely that the acid-regulation systems of *A. niger *were evolved in a medium as heavily buffered as a controlled bioreactor with pH regulation. It is thus efficient at a high pH to sacrifice biomass production for the production of large amounts of protons to reduce the pH quickly and to reduce this production at low pH values. We have not attempted to model this behavior, as there are very few available detailed data on acid production at different pH values. The work by Pedersen *et al. *[48] has sufficient detail for one level of pH, and thus this was used to approximate a constant ratio of protons to biomass. Although the assumption of a constant proton/biomass ratio is not valid over the full range of pH, it does allow us to study the simulated response in detail across the range of pH shown. Changing the proton/biomass ratio for individual pH value changes only the magnitude of the acid production and not the species.

In examining the transcriptional response, it was interesting - in the context of organic acids - to see that *oahA *and *goxC *are expressed and regulated, whereas no significant acid production occurs at the time of sampling for transcriptome analysis (Table 1). This suggests retention of the acid inside the cells or regulation of the gene product at a post-translational level.

In total, the number of genes influenced by ambient pH was surprisingly high. Although the transcriptional analysis is to some extent confounded by an effect on 'domestic' genes, the remaining response (2,022 genes) is still high. This response is not unlikely, as *A. niger *growing in nature acidifies the surroundings, thus living through a scale of pH values. This presumably requires a flexible and dynamic response of a large number of genes. Another point is, as Arst and Peñalva [49] correctly argue, when transcription of a gene is affected by ambient pH, this does not necessarily mean that it is regulated by pH. It may be an indirect consequence caused by differences in uptake efficiencies, intracellular metabolite levels, or other indirect effects. Most likely, a combination of the two is what we see here. For this purpose, the clustering and following identification of 109 genes with direct correlations with pH levels have proven to be a powerful method of data reduction.

The applied two-step clustering method allows differentiation between different effects, although it cannot determine which clusters of genes are directly or indirectly influenced by pH. One interesting application of this transcription study and the clustering is the prediction of regulatory motifs based on the transcription profiles. The predicted motifs are very likely to have the proposed function of increasing transcription with higher levels of pH, because one of the detected motifs was previously described to have this function. Although this, in theory, could be done for all of the 162 identified clusters, the performed predictions are limited to those described in the main matter of this study, but details on the clusters (see Additional data files 4 and 5) will support further investigation of other hypotheses. One obvious application of this is the identification of putative transcription factors regulated by ambient pH. We are currently constructing knockout strains for a large number of these.

The analysis of the clusters also includes the combination with data on the physical location of the genes. For the clusters predicted to be involved in secondary metabolite production, this physical location adds considerable value to the transcriptome analysis. It is confirmed that putative secondary metabolite clusters are transcriptionally regulated by pH. Even so, some of the identified co-regulated gene clusters may be artefacts, because of errors in predicting gene starts/stops. For example, if a gene erroneously has been predicted to be several genes, these will be seen as being co-regulated in the transcriptome analysis. Another likely explanation is that they are co-regulated by a common promoter region. However, for clusters of more than two genes, this is unlikely to be the case.

In a comparison of the modeling and the *in vivo *experiments, the predicted values correspond well with the profiles of oxalate production and the known literature. At all levels of pH, oxalate is a preferred acid (second to gluconate). As manganese was present in the medium and *oahA *was present in the strain, the funneling of carbon into citrate at lower pH could not be observed. As described in the work of Dai *et al. *[18] and Ruijter *et al. *[3], this is to be expected. Furthermore, when examining the transcription levels of *oahA*, which is found in subset E of Figure 4, they are on average 83 times higher at pH 2.5 compared with 6.0. This regulation counteracts citrate production. Thus, the predictions of the model are indeed valid, but the predicted (and known) optimum of citrate production are not replicated in the cultivations because of unknown factors.

The first steps toward understanding the pH-signaling pathway of *A. niger*, a pathway of great potential importance for the fermentation industry, are provided. The investigation of the *A. niger *homologue to the - in *A. nidulans* - well-described *pal *pH-signaling pathway showed that all components are present in *A. niger *and are expressed independent of pH. The uniqueness of this pathway makes it more than likely that these genes code for orthologues of the *A. nidulans *genes. However, a classic phenotypical characterization of mutants is still necessary to establish the function finally, but with this study, the targets for this characterization are now firmly established.

## Conclusions

We have shown through genome-scale modeling that the assumption of evolutionary selection for efficient acidification allows the reproduction of the pH optimum for production of citrate and oxalate by *A. niger*. Furthermore, our results indicate that high-yield gluconic acid production has not evolved as a trait for acidification of the growth habitat. Transcriptome analysis of *A. niger *cultures grown at three levels of pH showed 6,228 genes for which the transcription levels were significantly changed by direct and indirect effects of ambient pH. A two-step clustering method and GO term overrepresentation analysis identified 2,022 genes more likely to be influenced by pH and 109 genes with transcription levels directly corresponding to the level of pH. Analysis of these genes showed a strong co-regulation of *catR *and *goxA*. By combining genome coordinates with transcriptome profiles and predicted gene functions, secondary metabolite clusters found to be regulated by pH were identified. The *cis*-acting promoter motifs increasing transcription with higher levels of pH were identified, and a strategy for finding promoter motifs for other transcription profiles was presented. By using a combination of transcriptome data and sequence comparisons, the candidate orthologues of the *A. nidulans *Pal/PacC pH-regulation pathway were identified in *A. niger*. The conservation of this system supports that filamentous fungi have evolved to use several strategies for outcompeting rival organisms: an aggressive acidification of the microenvironment combined with storing the available glucose as gluconic acid.

## Materials and methods

### Modeling acid production

For each value of pH, a set of reactions was added to a genome-scale stoichiometric model of *A. niger *metabolism [7], thereby creating a model for each pH value. The reactions set consisted of seven reactions, one for each of the acids included in the model. Each reaction contains the fully protonated acid in an equilibrium with the partially unprotonated acid species and a number of protons. This number was calculated for each pH and acid by using the acid disassociation constant equation as shown in Equation 2:

(1)

(2)

In the case of polyprotic acids such as citric acid, a set of coupled equations - one for each acid group - was used. An example for citrate at pH 4.5 is shown in Equation 3 (see Additional data file 6 for the full set):

(3)

The entity CIT-e of Equation 3 is a mixed species, composed of citric acid molecules in various degrees of deprotonation, all in equilibrium at the given pH. It is assumed that the acids are transported across the cytoplasmic membrane fully protonated.

Modeling of acid production was performed by using stoichiometric matrices and linear programming for solving them, as described in reference [7]. Either the solving objective was a maximization of proton production with a fixed biomass production of 1 g or maximization for growth (growth-coupled proton production). For modeling of growth-coupled proton production, the biomass equation added a demand for 15.3 mmole of protons per gram of dry weight. This value was calculated from the oxalate and citrate yields of a pH 6.0 cultivation described by Pedersen *et al. *[48]. All simulations were performed with 100 mmole glucose and unlimited ammonium as substrates.

### Fermentation protocol

#### Strains

The strain used was *A. niger *BO-1, obtained from Novozymes A/S (Kalundborg, Denmark) and maintained as frozen spore suspensions at -80°C in 20% glycerol.

### Growth media

Complex medium: 2 g/L yeast extract, 3 g/L tryptone, 10 g/L glucose monohydrate, 20 g/L agar, 0.52 g/L KCl, 0.52 g/L MgSO_4_·7H_2_O, 1.52 g/L KH_2_PO_4 _and 1 ml/Lof trace elements solution. Trace element solution: 0.4 g/L CuSO_4_·5H_2_O, 0.04 g/L Na_2_B_4_O_7_·10H_2_O, 0.8 g/L FeSO_4_·7H_2_O, 0.8 g/L MnSO_4_·H_2_O, 0.8 g/L Na_2_MoO_4_·2H_2_O, 8 g/L ZnSO_4_·7H_2_O. Batch cultivation medium: 20 g/L glucose monohydrate, 2.5 g/L (NH_4_)_2_SO_4_, 0.75 g/L KH_2_PO_4_, 1.0 g/L MgSO_4_·7H_2_O, 1 g/L NaCl, 0.1 g/L CaCl_2_·2H_2_O, 0.05 ml/L antifoam 204 (Sigma-Aldrich, Brøndby, Denmark) and 1 ml/L trace element solution. Trace element solution composition: 7.2 g/L ZnSO_4_·7H_2_O, 0.3 g/L NiCl_2_·6H_2_O, 6.9 g/L FeSO_4_·7H_2_O, 3.5 g/L MnCl_2_·4H_2_O, and 1.3 g/L CuSO_4_·5H_2_O.

#### Preparation of inoculum

Fermentations were initiated by spore inoculation to a final concentration of 2 × 10^-9 ^spores/L. Spores were propagated on complex media plates and incubated for 7 to 8 days at 30°C before being harvested with 10 ml of 0.01% Tween 80.

#### Batch cultivations

Batch cultivations were performed in 2-L Braun fermenters with a working volume of 1.6 L, equipped with three Rushton four-blade disc turbines. The bioreactor was sparged with air, and the concentrations of oxygen and carbon dioxide in the exhaust gas were measured in a gas analyzer. The temperature was maintained at 30°C. The pH was controlled by automatic addition of 2 M NaOH. Agitation and aeration were controlled throughout the cultivations. For inoculation of the bioreactor, the pH was adjusted to 2.5; stirring rate, 100 rpm; and aeration, 0.1 volumes of air per volume of fluid per minute (vvm). After germination, the stirring rate was increased to 300 rpm, and the air flow, to 0.5 vvm. At 11 to 12 hours after inoculation, the stirring rate was increased to 600 rpm, and the air flow, to 1 vvm. When the CO_2 _in the exhaust gas reached a value of 0.1% (early growth phase), the stirring rate was set to 1,000 rpm. Additionally, at this level of CO_2_, the pH was slowly increased to 4.5 or 6.0 with a drop of 2 M NaOH every 10 seconds. For the cultivations at pH 2.5, pH was kept constant throughout the fermentation.

The concentrations of oxygen and carbon dioxide in the exhaust gas were monitored with a gas analyzer (1311 Fast response Triple gas, Innova combined with multiplexer controller for Gas Analysis MUX100, B. Braun Biotech International (Melsungen, Germany)).

#### Sampling

Cell dry weight was determined by using nitrocellulose filters (pore size, 0.45 μm; Pall Corporation, East Hills, NY, USA). The filters were predried in a microwave oven at 150 W for 15 minutes, cooled in a desiccator, and subsequently weighed. A known volume of cell culture was filtered, and the residue was washed with 0.9% NaCl and dried on the filter for 15 minutes in a microwave oven at 150 W and cooled in a desiccator. The filtrate was saved for quantification of sugars and extracellular metabolites and stored at -80°C. The filter was weighed again, and the cell mass concentration was calculated. These values were used to calculate maximal specific growth rates. For gene-expression analysis, mycelium was harvested at the mid to late exponential phase by filtration through sterile Mira-Cloth (Calbiochem, San Diego, CA, USA) and washed with phosphate-buffered saline (PBS) (8 g/L NaCl, 0.20 g/L KCl, 1.44 g/L Na_2_HPO_4_, and 0.24 g/L KH_2_PO_4 _in distilled water). The mycelium was quickly dried by squeezing, and subsequently frozen in liquid nitrogen. Samples were stored at -80°C until RNA extraction.

#### Quantification of sugars and extracellular metabolites

The concentrations of sugar and organic acids in the filtrates were determined by using HPLC on an Aminex HPX-87H ion-exclusion column (BioRad, Hercules, CA, USA). The column was eluted at 60°C with 5 mM H_2_SO_4 _at a flow rate of 0.6 ml/min. Metabolites were detected with a refractive index detector and a UV detector.

#### Calculation of maximum specific growth rates

The maximal growth rate was determined by performing exponential regressions on the data points from the exponential phase (defined as the part of the growth curve that exhibited linear increase in a single-log plot) for each of the nine experiments. Means and standard deviations were calculated for each set of triplicates.

### Transcriptome analysis

#### Extraction of total RNA

From 40 to 50 mg of frozen mycelium was placed in a 2-ml Eppendorf tube, precooled in liquid nitrogen, containing three steel balls (two balls with a diameter of 2 mm and one ball with a diameter of 5 mm). The tubes were then shaken in a Mixer Mill, at 5°C for 10 minutes, until the mycelia were ground to powder. Total RNA was isolated from the powder by using the Qiagen RNeasy Mini Kit, according to the protocol for isolation of total RNA from plant and fungi. The quality of the extracted total RNA was assessed by using a BioAnalyzer 2100 (Agilent Technologies, Inc., Santa Clara, CA, USA), and the quantity determined by using a spectrophotometer (GE Healthcare Bio-Sciences AB, Uppsala, Sweden). The total RNA was stored at -80°C until further processing.

#### Preparation of biotin-labeled cRNA and microarray processing

Fragmented biotin-labeled cRNA (15 μg) was prepared from 5 μg of total RNA and hybridized to the 3AspergDTU GeneChip [50] according to the Affymetrix GeneChip Expression Analysis Technical Manual [51].

cRNA was quantified in a spectrophotometer (same as described earlier). cRNA quality was assessed by using a BioAnalyzer. A GeneChip Fluidics Station FS-400 (fluidics protocol FS450_001) and a GeneChip Scanner 3000 were used for hybridization and scanning.

The scanned probe array images (.DAT files) were converted into .CEL files by using the GeneChip Operating Software (Affymetrix).

#### Analysis of transcription data

Affymetrix CEL-data files were preprocessed by using the statistical language and environment R [52] version 2.5.1. The probe intensities were normalized for background by using the robust multiarray average (RMA) method [53] with only perfect match (PM) probes. Normalization was performed subsequently by using the quantiles algorithm [54]. Gene-expression values were calculated from the PM probes with the median polish summary method [53]. All statistical preprocessing methods were used by invoking them through the Affy package [55].

Statistical analysis was applied to determine genes subject to differential transcriptional regulation. The limma package [56] was used to perform moderated *t *tests between two sets of triplicates from each pH level. Empiric Bayesian statistics were used to moderate the standard errors within each gene, and Benjamini-Hochberg's method [57], to adjust for multitesting. A cut-off value of adjusted *P *< 0.05 was set to assess statistical significance.

Normalized and raw data values are deposited with Gene Expression Omnibus [GEO:GSE11725].

### Clustering

The 6,228 genes that showed significant changes in expression indices in one or more pairwise comparisons were sorted into groups based on the direction of their response in the three different sets of conditions. These groups were divided into a varying number of subgroups (clusters) by using the clustering algorithm ClustreLustre [58], with k-means normalization. The groups were divided until all clusters had a minimum separation distance of 1.01. This number was picked empirically and was the minimal distance at which each cluster still had a distinct regulation pattern.

### Identification of co-regulated gene clusters

Co-regulated gene clusters were defined in this study as genes on the same scaffold that are separated by no more than 5 kilobases, significantly regulated in one or more pairwise comparisons, and having the same regulation pattern as determined by the detailed clustering by using ClusterLustre.

### Prediction of conserved motifs

Conserved motifs were predicted by using R 2.6.2 [52] with the Cosmo package v. 1.4.0 [59]. Default settings were used with the following exceptions: A background Markov model was computed by using the intergenic regions from scaffold 1 of the *A. niger *ATCC 1015 genome sequence. Intergenic regions containing unknown bases (Ns) were pruned from the training set, amounting to 1.7 Mb in 1,214 sequences. The Two-Component-Mixture (TCM), One Occurrence Per Sequence (OOPS) and Zero Or One Occurrence Per Sequence (ZOOPS) motif models were used to search for conserved motifs. For all query sequences 1,000 base pairs upstream of the start codon of the gene or of the predicted transcription start were, if any was found. If a transcription start was predicted, the sequence from this base pair and to the start codon was included as well, thus increasing the length of sequence to more than 1 kilobase.

### GO-term enrichment analysis

Significantly regulated subsets of genes were examined for GO-term enrichment by using R-2.5.1 [52] with BioConductor [60] and the topGO-package v. 1.2.1 by using the elim algorithm to remove local dependencies between GO terms [61]. GO-term assignments were based on automatic annotation of the *A. niger *ATCC 1015 v1.0 gene models. Where nothing else is noted, *P *< 0.05 is used as the cutoff for significance.

## Abbreviations

JGI: Joint Genome Institute; ORFs: open reading frames; PBS: phosphate-buffered saline; ppb: parts per billion; vvm: volumes of air per volume of fluid per minute.

## Authors' contributions

MRA wrote the manuscript, analyzed data, designed the experiments, and performed a part of the microarray hybridization. LL wrote the expansion of the model, analyzed data, and performed bioreactor cultivations and the majority of the microarray hybridization. JN designed the experiments and supervised the work.

## Additional data files

The following additional data are available with the online version of this article. Additional data file 1 is a figure containing an overview of modeled acid production as a function of pH maximizing for growth coupled with acid (proton) production. Additional data file 2 is a figure containing an overview of modeled acid production as a function of pH, maximizing for proton production with fixed growth. Additional data file 3 is a text file with GO term overrepresentation results for all clusters of Additional data file 5 except cluster D, which has only one gene. For each of the three ontologies (metabolic function, biologic process, and cellular component) is shown the significant terms (p.elim < 0.05). Additional data file 4 is a figure showing the clusters of genes co-regulated by ambient pH. Cluster D is not shown, as it contains only one gene. The clusters are grouped based on statistical significance in pairwise comparisons of transcriptome data at pH values of 2.5, 4.5, and 6.0. Each cluster has nine values. The first three are biologic replicates at pH 2.5; the middle three are at pH 4.5; and the last three are from pH 6.0. The genes are clustered by using Matlab and the ClustreLustre algorithm [58]. Additional data file 5 is a table containing the clustering of *A. niger *genes with significantly changed expression indices. The HiLo, MeLo, and HiMe columns are log2 ratios of gene-expression indices in comparisons of pH 6.0 (Hi), pH 4.5 (Me), and pH 2.5 (Lo). Negative values mean that the index was higher in the second condition. If the comparison was not significant, a N/A is shown. Annotations are manually extracted from the Joint Genome Institute (JGI) website [62] and are the manual annotation (if that was present), or a general Interpro or GO-term prediction (if no manual annotation was present). A '-' means that no putative function could be assigned. The Colocalized column: Genes with the same number are colocalized. The Sec Metabolites column: Genes that are believed to be in a colocalized, co-regulated secondary metabolite cluster are marked with numbers. Additional data file 6 is a table of the proton-creating reactions of the acid-production model that were added to the previously described genome-scale stoichiometric model of *A. niger *[7].

## Supplementary Material

Additional file 1 Figure containing an overview of modeled acid production as a function of pH maximizing for growth coupled with acid (proton) production. Click here for file

Additional file 2 Figure containing an overview of modeled acid production as a function of pH, maximizing for proton production with fixed growth. Click here for file

Additional file 3 For each of the three ontologies (metabolic function, biologic process, and cellular component) is shown the significant terms (p.elim < 0.05). Click here for file

Additional file 4 Cluster D is not shown, as it contains only one gene. The clusters are grouped based on statistical significance in pairwise comparisons of transcriptome data at pH values of 2.5, 4.5, and 6.0. Each cluster has nine values. The first three are biologic replicates at pH 2.5; the middle three are at pH 4.5; and the last three are from pH 6.0. The genes are clustered by using Matlab and the ClustreLustre algorithm [58]. Click here for file

Additional file 5 The HiLo, MeLo, and HiMe columns are log2 ratios of gene-expression indices in comparisons of pH 6.0 (Hi), pH 4.5 (Me), and pH 2.5 (Lo). Negative values mean that the index was higher in the second condition. If the comparison was not significant, a N/A is shown. Annotations are manually extracted from the Joint Genome Institute (JGI) website [62] and are the manual annotation (if that was present), or a general Interpro or GO-term prediction (if no manual annotation was present). A '-' means that no putative function could be assigned. The Colocalized column: Genes with the same number are colocalized. The Sec Metabolites column: Genes that are believed to be in a colocalized, co-regulated secondary metabolite cluster are marked with numbers. Click here for file

Additional file 6 Table of the proton-creating reactions of the acid-production model that were added to the previously described genome-scale stoichiometric model of *A. niger *[7]. Click here for file
