# Supplementary figures and images for: Systemic analysis of the response of Aspergillus niger to ambient pH
Source: Genome Biol. 2009 May 1;10(5):R47. doi: 10.1186/gb-2009-10-5-r47 (PMC2718513; doi:10.1186/gb-2009-10-5-r47)

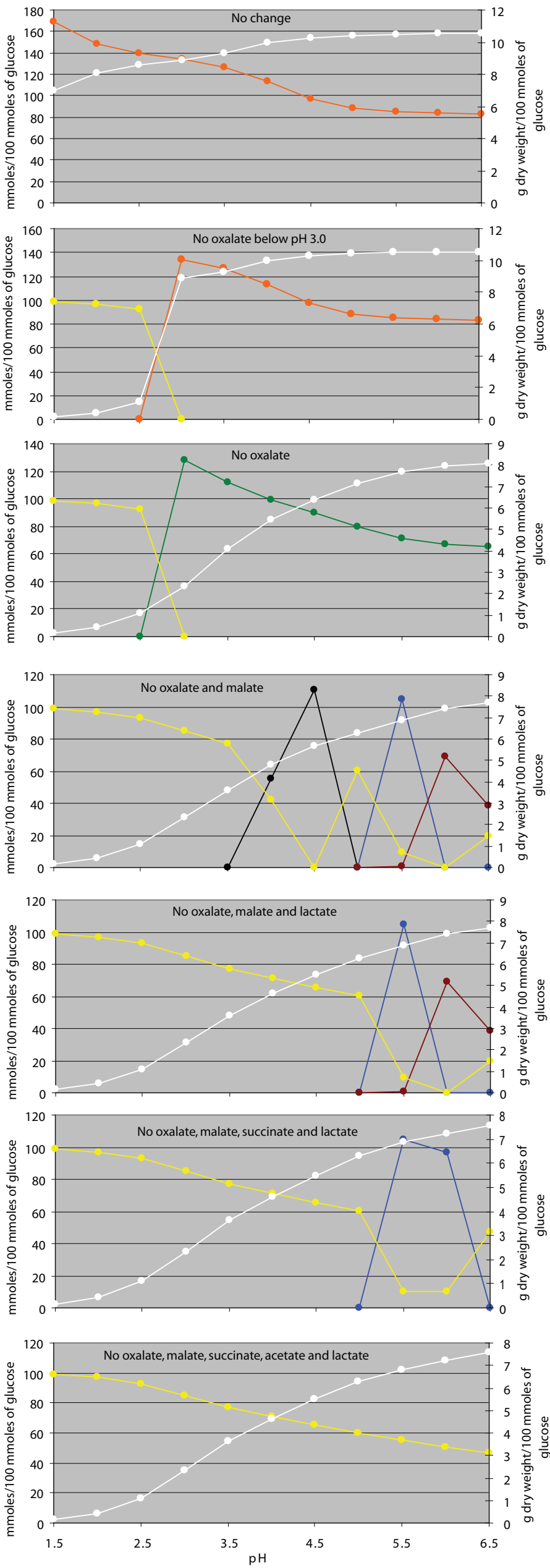

Protons      Gluconate      Oxalate      Citrate  
Lactate      Succinate      Acetate      Malate

Supplement: Additional file 1 — Figure containing an overview of modeled acid production as a function of pH maximizing for growth coupled with acid (proton) production. [file gb-2009-10-5-r47-S1.pdf]

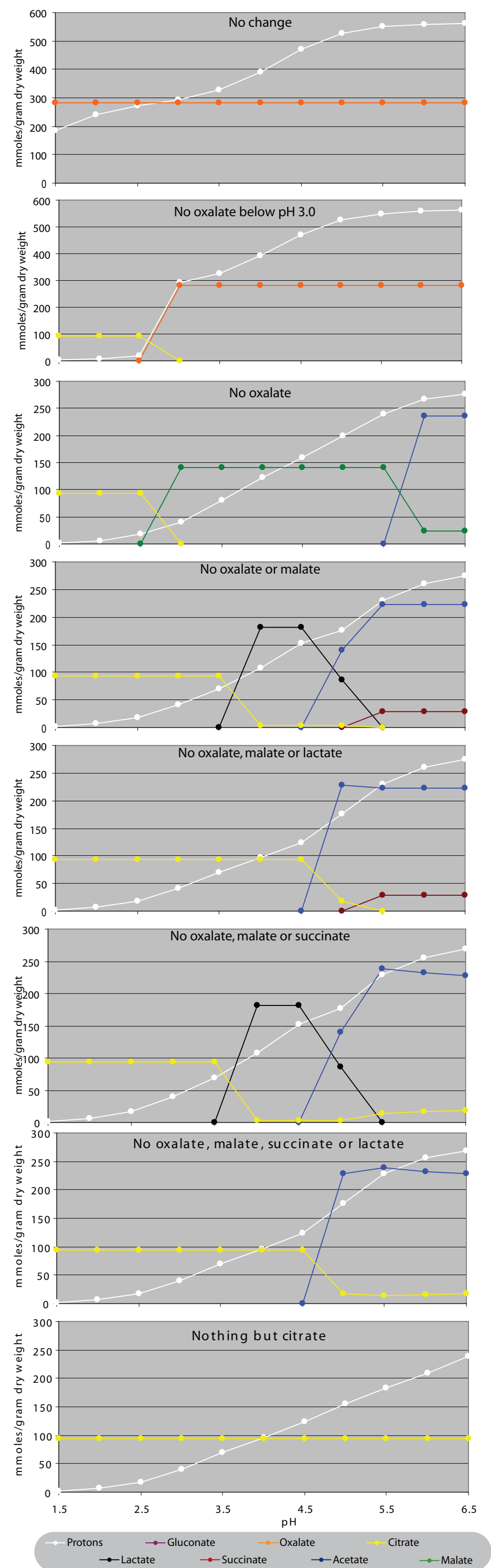

Supplement: Additional file 2 — Figure containing an overview of modeled acid production as a function of pH, maximizing for proton production with fixed growth. [file gb-2009-10-5-r47-S2.pdf]

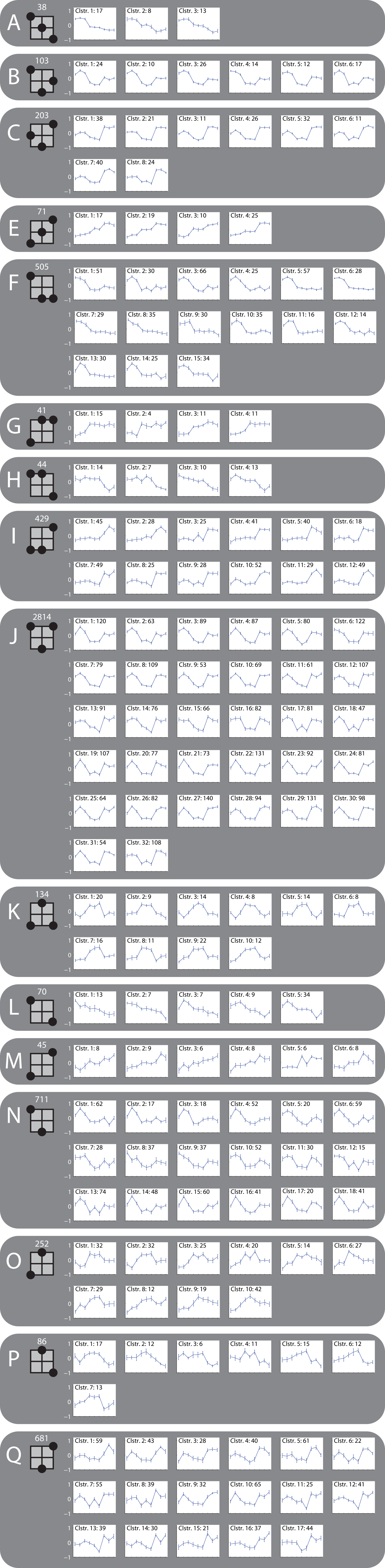

Supplement: Additional file 4 — Cluster D is not shown, as it contains only one gene. The clusters are grouped based on statistical significance in pairwise comparisons of transcriptome data at pH values of 2.5, 4.5, and 6.0. Each cluster has nine values. The first three are biologic replicates at pH 2.5; the middle three are at pH 4.5; and the last three are from pH 6.0. The genes are clustered by using Matlab and the ClustreLustre algorithm [58]. [file gb-2009-10-5-r47-S4.pdf]
