# Supplementary material for: Systemic analysis of the response of Aspergillus niger to ambient pH
Source: Genome Biol. 2009 May 1;10(5):R47. doi: 10.1186/gb-2009-10-5-r47 (PMC2718513; doi:10.1186/gb-2009-10-5-r47)
Supplement: Additional file 6 — Table of the proton-creating reactions of the acid-production model that were added to the previously described genome-scale stoichiometric model of A. niger [7]. [file gb-2009-10-5-r47-S6.pdf]

Suppl. Table 6: Proton-creating reactions of the acid-production model

pH	1.5	2	2.5	3
Citrate	CITe ⇌ CIT-e + 0.0224 H+e	CITe ⇌ CIT-e + 0.0677 H+e	CITe ⇌ CIT-e + 0.1882 H+e	CITe ⇌ CIT-e + 0.4313 H+e
Gluconate	GLCNTe ⇌ GLCNT-e + 0.0063 H+e	GLCNTe ⇌ GLCNT-e + 0.0196 H+e	GLCNTe ⇌ GLCNT-e + 0.0594 H+e	GLCNTe ⇌ GLCNT-e + 0.1663 H+e
Lactate	LACe ⇌ LAC-e + 0.0043 H+e	LACe ⇌ LAC-e + 0.0136 H+e	LACe ⇌ LAC-e + 0.0418 H+e	LACe ⇌ LAC-e + 0.1213 H+e
Acetate	ACe ⇌ AC-e + 0.0006 H+e	ACe ⇌ AC-e + 0.0018 H+e	ACe ⇌ AC-e + 0.0056 H+e	ACe ⇌ AC-e + 0.0175 H+e
Oxalate	OXALe ⇌ OXAL-e + 0.6524 H+e	OXALe ⇌ OXAL-e + 0.8611 H+e	OXALe ⇌ OXAL-e + 0.969 H+e	OXALe ⇌ OXAL-e + 1.044 H+e
Succinate	SUCCe ⇌ SUCC-e + 0.0022 H+e	SUCCe ⇌ SUCC-e + 0.0069 H+e	SUCCe ⇌ SUCC-e + 0.0214 H+e	SUCCe ⇌ SUCC-e + 0.0651 H+e
Malate	MALe ⇌ MAL-e + 0.0124 H+e	MALe ⇌ MAL-e + 0.0383 H+e	MALe ⇌ MAL-e + 0.1124 H+e	MALe ⇌ MAL-e + 0.2885 H+e
pH	3.5	4	4.5	5
Citrate	CITe ⇌ CIT-e + 0.7431 H+e	CITe ⇌ CIT-e + 1.0253 H+e	CITe ⇌ CIT-e + 1.3196 H+e	CITe ⇌ CIT-e + 1.6553 H+e
Gluconate	GLCNTe ⇌ GLCNT-e + 0.3869 H+e	GLCNTe ⇌ GLCNT-e + 0.6661 H+e	GLCNTe ⇌ GLCNT-e + 0.8632 H+e	GLCNTe ⇌ GLCNT-e + 0.9523 H+e
Lactate	LACe ⇌ LAC-e + 0.3039 H+e	LACe ⇌ LAC-e + 0.5799 H+e	LACe ⇌ LAC-e + 0.8136 H+e	LACe ⇌ LAC-e + 0.9324 H+e
Acetate	ACe ⇌ AC-e + 0.0532 H+e	ACe ⇌ AC-e + 0.151 H+e	ACe ⇌ AC-e + 0.3599 H+e	ACe ⇌ AC-e + 0.6401 H+e
Oxalate	OXALe ⇌ OXAL-e + 1.1644 H+e	OXALe ⇌ OXAL-e + 1.3909 H+e	OXALe ⇌ OXAL-e + 1.6709 H+e	OXALe ⇌ OXAL-e + 1.8659 H+e
Succinate	SUCCe ⇌ SUCC-e + 0.1821 H+e	SUCCe ⇌ SUCC-e + 0.4247 H+e	SUCCe ⇌ SUCC-e + 0.7528 H+e	SUCCe ⇌ SUCC-e + 1.0726 H+e
Malate	MALe ⇌ MAL-e + 0.5768 H+e	MALe ⇌ MAL-e + 0.8694 H+e	MALe ⇌ MAL-e + 1.1253 H+e	MALe ⇌ MAL-e + 1.4170 H+e
pH	5.5	6	6.5	
Citrate	CITe ⇌ CIT-e + 1.9552 H+e	CITe ⇌ CIT-e + 2.2374 H+e	CITe ⇌ CIT-e + 2.5503 H+e	
Gluconate	GLCNTe ⇌ GLCNT-e + 0.9844 H+e	GLCNTe ⇌ GLCNT-e + 0.995 H+e	GLCNTe ⇌ GLCNT-e + 0.9984 H+e	
Lactate	LACe ⇌ LAC-e + 0.9776 H+e	LACe ⇌ LAC-e + 0.9928 H+e	LACe ⇌ LAC-e + 0.9977 H+e	
Acetate	ACe ⇌ AC-e + 0.849 H+e	ACe ⇌ AC-e + 0.9468 H+e	ACe ⇌ AC-e + 0.9825 H+e	
Oxalate	OXALe ⇌ OXAL-e + 1.9533 H+e	OXALe ⇌ OXAL-e + 1.9847 H+e	OXALe ⇌ OXAL-e + 1.9951 H+e	
Succinate	SUCCe ⇌ SUCC-e + 1.4010 H+e	SUCCe ⇌ SUCC-e + 1.7033 H+e	SUCCe ⇌ SUCC-e + 1.8849 H+e	
Malate	MALe ⇌ MAL-e + 1.7066 H+e	MALe ⇌ MAL-e + 1.8853 H+e	MALe ⇌ MAL-e + 1.9609 H+e	
